# Analysis of the main characteristics of children's skin moisturizers in the Brazilian market^☆^

**DOI:** 10.1016/j.jped.2025.101438

**Published:** 2025-09-20

**Authors:** Bruna Rocha Reolon Branchi, Ana Elisa Kiszewski, Renan Rangel Bonamigo

**Affiliations:** a Universidade de Ciências da Saúde de Porto Alegre (UFCSPA), Porto Alegre, RS, Brazil; b Universidade de Passo Fundo (UPF), Passo Fundo, RS, Brazil; c Universidade Federal do Rio Grande do Sul (UFRGS), Porto Alegre, RS, Brazil; d Hospital de Clínicas de Porto Alegre (HCPA), Porto Alegre, RS, Brazil

**Keywords:** Emollients, Cosmetics, Hydrogen-ion concentration, Microbiota, Child, Pediatrics

## Abstract

**Objective:**

One of the possible causes of skin microbiome imbalance is the use of dermocosmetics with inadequate pH. This study aims to critically evaluate several children's moisturizers regarding their characteristics so that we can verify the tendency of the products available on the market and whether they are slightly acidic. The importance of dermocosmetics formulated without ingredients with allergenic potential is also discussed in this work.

**Method:**

Observational, analytical, cross-sectional and quantitative study. Several brands of children's moisturizers were selected and divided into two groups: group 1 (G1), with moisturizers focused on the care of children with normal skin; and group 2 (G2), with moisturizers with a therapeutic focus on atopic children. We analyzed the pH of each one of the moisturizers, as well as cost, presence of potentially allergenic components and other data contained in the packaging.

**Results:**

The members of G1 had an average pH of 5.81 ± 0.35, while the members of G2 had an average pH of 5.42 ± 0.28, with this difference being considered statistically significant (*p* ≤ 0.001). G1 differed in terms of cost, which was more affordable for the user, when compared to G2 (*p* ≤ 0.001), but with a predominance of potential allergens in its composition (*p* = 0.018).

**Conclusion:**

This study demonstrates that all moisturizers analyzed in this study respected the acidic pH; however, the group of moisturizers with a therapeutic focus on atopic children had an even lower pH and lower allergenic potential in their composition compared to the group of moisturizers focused on care of children with normal skin.

## Introduction

Skin pH is a central regulator of skin barrier homeostasis and an important innate defense mechanism, being considered an important protective factor against microorganisms, essential for the maturation of the epidermal barrier and for repair processes.^1^, ^2^, ^3^, ^4^ Different bacterial species can grow on human skin depending on the pH of its surface, and a balance of these microorganisms is necessary to achieve healthy skin. At birth, a full-term newborn has a skin pH that ranges from 6.3 to 7.5. Within the first two weeks of life, the pH drops to approximately 5. Between the second and fourth weeks of life, the pH gradually becomes acidic, ranging from 4.2 to 5.9, depending on the area of ​​the body. In adults and adolescents, the skin pH is <5.^2^, ^3^, ^4^, ^5^, ^6^, ^7^, ^8^, ^9^, ^10^ Compared to healthy control subjects, patients diagnosed with atopic dermatitis exhibit a skin surface pH that is between 0.1 and 0.9 pH units higher.^11^

The literature recommends the use of dermocosmetics with acidic pH since they do not interfere as intensely with the skin microflora. These products have become an option in dermatological diseases related to xerosis, such as atopic dermatitis and, according to individualized assessment, even for healthy skin.^1^^,^^3^, ^4^, ^5^, ^6^, ^7^, ^8^, ^9^, ^10^, ^11^, ^12^, ^13^, ^14^, ^15^, ^16^

Since bathing can dehydrate the stratum corneum, moisturizers are helpful in rehydrating the skin.^5^^,^^17^ Moisturizers are usually produced in lotion or cream vehicles but may also be available in other vehicles – such as baume,^18^ mist, and gel cream. For the chosen skin moisturizers, products with fragrance, preservatives, and sensitizing substances should be avoided, considering each patient's preference regarding the texture in lotion or cream and the cost of the product, which will be used daily, and in some patients several times a day, and over the years.^17^

The main allergens described as possibly present in children's dermocosmetics are fragrance/perfume, propylene glycol, lanolin, methylisothiazolinone (MI), cocamidopropyl betaine, and formaldehyde, which are considered the most relevant according to recent literature for the risk of contact dermatitis.^19^, ^20^, ^21^, ^22^ Associated with these allergens are others with emerging allergenic potential, such as pentylene glycol.^23^^,^^24^ Another subject in the composition of dermocosmetics is the presence of potentially allergenic olfactory notes, which must be described separately on the labels according to national legislation, in addition to the description of essence, perfume and/or fragrance present as an ingredient in the formulation, when its concentration exceeds 0.001 % in leave-in products and 0.01 % in rinse-off products.^25^

A relevant concept is about the chemical endocrine disruptors, which are defined as an exogenous substance or mixture that alters the function of the endocrine systems – which can lead to endocrine disorders such as diabetes, obesity, thyroid disease, and changes in adrenal hormones – and, consequently, causes adverse effects in an intact organism or its progeny. The main chemical compounds identified as potential endocrine disruptors are phthalates, PFAS (perfluoroalkyl and polyfluoroalkyl substances), Bisphenol A (BPA), triclosan, parabens, and phenols.^26^^,^^27^ They are often present in cosmetics, personal hygiene products, and other scented household items.^26^ We emphasize the importance of children's cosmetics being formulated without endocrine disruptors nowadays to avoid an increased potential risk of endocrine disorders that may develop.

Thus, this study critically evaluates several children's moisturizers regarding their characteristics – pH and composition, information available on the packaging – so that we can verify the trend of the products available on the market, whether they are slightly acidic, and whether they are consistent with maintaining the lipid mantle and the skin's barrier function. The study's application focuses on care for children with normal skin and therapeutic care for atopic children.

## Methods

### Observational, analytical, cross-sectional, and quantitative study

Several brands and models of children's moisturizers were selected between the period of April 1, 2024 and June 30, 2024, and divided into two main groups: group 1 (G1), with brands available in supermarkets, pharmacies and physical or online cosmetics stores with marketing to the general public and focused on serving children with normal skin; and group 2 (G2), with moisturizers with a therapeutic focus on atopic children, generally sold with a prescription and/or guidance from a pediatrician or dermatologist with marketing aimed at them.

The moisturizers were selected from all those that contained on the label some reference that they can be used for children and that were available for purchase in person within a 5 km radius of the Universidade de Ciências da Saúde de Porto Alegre (UFCSPA) or through the main online shopping sites such as Panvel, Droga Raia, São João, Boticário, Avon, Granado, and Natura.

The variables evaluated were the following: pH of each moisturizer, types of vehicles used (lotion, cream or others), cost, presence of potentially allergenic components, presence of fragrance, dyes, parabens or other potential endocrine disruptors, and data contained on the packaging – such as ideal pH for children's skin or acidic pH, dermatologically tested or hypoallergenic, reference to sensitive skin, reference to sustainability and/or recycling, cruelty free, presence of natural and/or vegan ingredients.

The moisturizers were placed in containers, and the formulations were measured in triplicate by directly measuring the pH using a Gehaka® PG2000 benchtop pH meter (initially calibrated with standard pH solutions of pH 4.0 and 6.86). The data obtained were recorded in a Microsoft Excel® spreadsheet. After obtaining the results, the products were grouped according to pH and subdivided into 3 categories by pH range: <5; between 5 and 5.9; and above 6. We then performed a descriptive analysis of the exposed data.

Regarding statistical analysis, the program used for statistical analyses was the Statistical Package for the Social Sciences, version 24.0 (SPSS®), with PSM (Propensity Score Matching) extension, commercially available and freely accessible. Microsoft Excel® was used for graphs. Descriptive statistics of categorical variables were shown in absolute numbers and percentages, and of numerical variables, mean (standard deviation) and median (interquartile range), according to a previous assessment to define the behavior of numerical variables using the Kolmogorov-Smirnov normality test. Fisher's exact test was used for analytical statistics to compare proportions, Student's *t*-test for independent samples was used to compare means, and the Mann-Whitney U test was used to compare medians. The minimum significance level or probability of significance adopted was 5 % on both sides.

## Results

**Figure 1:**
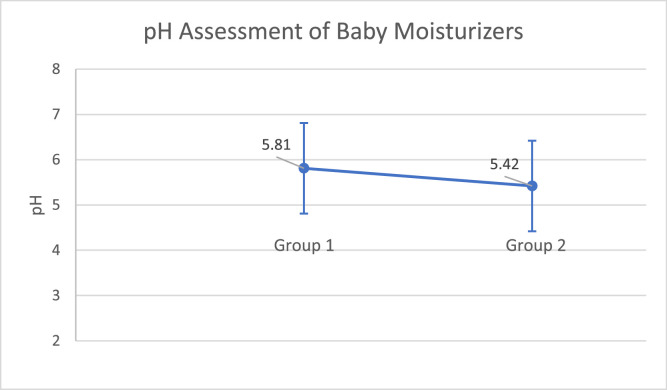
Difference in pH of moisturizers between groups 1 and 2, with their respective means and standard deviation (*p* ≤ 0.001).

The pH of the moisturizers ranged from 4.87 to 6.50, with an average of 5.81 (±0.35) for the moisturizers in group 1 and 5.42 (±0.28) for those in group 2, as shown in Table 1. When comparing the data, it was observed that in relation to the type of moisturizer between group 1 and group 2, there was a statistically significant difference (*p* ≤ 0.001), confirming the hypothesis that the moisturizers in group 2 presented lower pH values ​​in this study, as illustrated in Figure 1.

**Table 1:** Brands of baby moisturizers selected in this sample divided into two groups and their respective hydrogen potential (pH) values.

**Selected brand/model of baby moisturizers**	**pH**
<**5**	
**Group 1**	
None	
**Group 2**	
Klaviê Clinical Loção Hidratante Adulto e Infantil Theraskin	4.87
Hidratante Nutritivo Derma Protect Johnson’s Baby	4.90
Loção Hidratante Cetaphil Pro AD Restoraderm	4.87
**Between 5 and 5.9**	
**Group 1**	
Loção Hidratante Infantil Panvel Baby	5.40
Loção Corporal Hidratação Relaxante Dove Baby Hora de Dormir	5.62
Loção Hidratante Dove Baby Hidratação Enriquecida	5.44
Loção Hidratante Corporal Mustela Hydra Bebê	5.64
Creme Hidratante Mustela Bio	5.10
Loção Hidratante Pampers Girassol	5.78
Loção Hidratante Pampers Babytopia	5.62
Creme Hidratante Reparador Pampers	5.75
Loção Hidratante Johnson’s Baby Uso Diário	5.40
Loção Hidratante Johnson’s Baby Hora do Sono	5.30
Loção Cremosa Johnson’s Baby Hidratação Intensa	5.68
Creme Corporal Pele Delicada Chicco Baby Moments	5.42
Loção Hidratante Natura Mamãe E Bebê	5.70
Creme Reparador Hidratante Cicababy Boti Baby Boticário	5.67
Loção Hidratante Corporal Lavanda Needs Baby	5.42
Loção Banho e Pós Banho Boti Baby Nana Neném Boticário	5.72
Loção Glitter Hidratante Sophie Wish O Boticário	5.60
**Group 2**	
Loção Hidratante Umiditá Infantil Pele Sensível	5.56
Loção Hidratante Infantil Hidra Kids D.A. Pele Sensível	5.40
Hidratante Calmante Para Pele Muito Sensível Mustela	5.62
Hidratante Stelatopia + Relipidante Mustela	5.60
Loção Intensiva Corporal Hidratante Neutrogena Norwegian A48	5.34
Loção Hidratante CeraVe	5.45
Creme Hidratante CeraVe	5.56
Loção Hidratante Hydraporin AI	5.42
Bruma Corporal Hidratante Hydraporin AI	5.58
Creme AI Fisiogel Para Pele Seca, Sensível E Irritada	5.72
Bálsamo Lipídico Restaurador AI Fisiogel Para Pele Seca, Sensível E Irritada	5.02
Gel Creme Eucerin pH 5	5.58
Loção Hidratante Corporal Isdin Para Pele Atópica Nutratopic Pro-AMP	5.75
Bioderma Atoderm Intensive Gel Creme	5.31
Bioderma Atoderm Intensive Baume	5.72
Creme Reparador Avene Cicalfate+	5.30
Hidratante Corporal La Roche Posay Lipikar Baume Light AP+*M*	5.60
Cicaplast Baume B5 La Roche Posay Multirreparador	5.27
NutriolMed Hidratante Intensivo Anticoceira Darrow	5.70
**Above 6**	
**Group 1**	
Creme Reparador Mustela Cicastela	6.20
Loção Hidratante Johnson’s Baby Recém Nascido	6.15
Loção Hidratante Infantil Granado Bebê Tradicional	6.00
Loção Hidratante Infantil Granado Bebê Camomila	6.15
Loção Hidratante Infantil Granado Bebê Calêndula	6.15
Loção Hidratante Infantil Granado Bebê Erva Doce	6.12
Loção Hidratante Infantil Granado Bebê Lavanda	6.20
Loção Hidratante Dermocalmante Granado Bebê Peles Sensíveis	6.00
Loção Hidratante Relaxante Natura Mamãe E Bebê	6.26
Loção Hidratante Lero-Lero Natura Naturé	6.02
Loção Hidratante Para O Corpo Calming Avon Care Baby	6.37
Loção Hidratante Banho e Pós-Banho Boti Baby Boticário	6.50
**Group 2**	
None

In group 1, we analyzed a total of 29 moisturizers, while in group 2, 22 moisturizers were analyzed. Three moisturizers in group 2 had a pH < 5.0 (13.6 %), and no moisturizer in group 1 was in this pH range. When we analyzed the moisturizers with a pH between 5 and 5.9, we observed that we obtained 17 from group 1 (58.6 %) and 19 from group 2 (86.4 %). Twelve moisturizers in group 1 had a pH ≥ 6.0 (41.4 %), in contrast to no moisturizer in group 2 in this pH range. There was no moisturizer with a pH > 7 in this sample.

When we analyzed the type of vehicle used in the formulation of the moisturizers, we could observe a predominance of the lotion form in both groups, but mainly in group 1, where 24 moisturizers were presented with a lotion vehicle, and in second place, 5 moisturizers with a cream vehicle. In group 2, we could observe a greater frequency of the lotion form with 10 moisturizers, in second place, 7 moisturizers in the other category – such as gel cream, balm and mist – and in third place, 5 moisturizers with a cream vehicle. This comparison analysis using the Student's *t*-test for independent samples showed statistical significance (*p* = 0.002).

When analyzing the cost of moisturizers, we chose to separate them into two categories – one for the cost of the smallest packaging available on the market and the other for the cost per 100 mL of moisturizer in the largest packaging available. When we analyzed the cost for the smallest packaging available, we observed that the cost of moisturizers in group 1 had a median of R$43.00 (R$ 29.50–58.40) and group 2 of R$ 88.00 (R$ 67.50–128.00), with statistical significance (*p* ≤ 0.001). Likewise, when we analyzed the cost per 100 mL of moisturizer, group 1 had a median of R$ 24.60 (R$ 18.50–33.40) and group 2 of R$70.40 (R$ 44.00–80.00), also with statistical significance (*p* ≤ 0.001).

The data described below are available in Table 2. We analyzed the items contained in the moisturizer packaging, in which we were able to assess that almost all of them were indicated as being dermatologically tested or hypoallergenic in both groups (G1 100 %/G2 90.9 %) and most did not contain dyes (G1 96.6 %/G2 95.5 %) or parabens in their composition (G1 96.6 %/G2 100 %), with no statistically significant difference between the groups. We also demonstrated the absence of other potential endocrine disruptors in the sample, since none of them contained phthalates or phenols in their composition.

**Table 2:** Analysis of the composition of the various children's moisturizers selected in this sample, as well as of items present in the product packaging, separated into group 1 and group 2.

**Variables**	**Moisturizer groups**		**p-value**
	**Group 1**	**Group 2**	
Dermatologically tested or hypoallergenic	29 (100 %)	20 (90,9 %)	0181
Sensitive skin	7 (24,1 %)	15 (68,2 %)	0004*
Fragrance free	7 (24,1 %)	20 (90,9 %)	≤ 0001*
No dyes	28 (96,6 %)	21 (95,5 %)	1,00
Absence of endocrine disruptors Paraben-free Phthalate-free Phenol-free	28 (96,6 %) 29 (100 %) 29 (100 %)	22 (100 %) 22 (100 %) 22 (100 %)	1,00 1,00 1,00
Reference to natural ingredients	20 (69 %)	5 (22,7 %)	0002*
Vegan	15 (51,7 %)	2 (9,1 %)	0002*
Reference to sustainability and/or recycling	13 (44,8 %)	4 (18,2 %)	0072
Cruelty free	13 (44,8 %)	3 (13,6 %)	0031*
pH reference on packaging	9 (31 %)	3 (13,6 %)	0192
Presence of possible allergens Fragrance Propylene glycol Lanolin Methylisothiazolinone Cocamidopropyl betaine Formaldehyde Pentilene glycol Olfactory notes	23 (79,3 %) 22 (75,9 %) 3 (10,3 %) 0 (0 %) 0 (0 %) 0 (0 %) 0 (0 %) 0 (0 %) 4 (13,8 %)	10 (45,5 %) 2 (9,1 %) 2 (9,1 %) 0 (0 %) 0 (0 %) 2 (9,1 %) 0 (0 %) 6 (27,3 %) 0 (0 %)	0018* ≤ 0001* 1,00 1,00 1,00 0181 1,00 0004* 0124

* With statistical significance (Fisher's Exact Test).

Moisturizers that contained a reference to sensitive skin on the packaging were mostly part of group 2 (G1 24.1 %/G2 68.2 %; *p* = 0.004). As well as in the evaluation regarding the absence of fragrance or perfume in the components of the moisturizers, in which we were able to evaluate a statistically significant difference, with a predominance of group 2 for this analysis (G1 24.1 %/G2 90.9 %; *p* ≤ 0.001).

However, in terms of the presence of natural ingredients in the composition of moisturizers, we observed a predominance of these in group 1 (G1 69 %/G2 22.7 %; *p* = 0.002), as well as in the evaluation of vegan ingredients (G1 51.7 %/G2 9.1 %; *p* = 0.02), reference to sustainability and/or recycling (G1 44.8 %/G2 18.2 %; *p* = 0.072) and products referred as cruelty-free (G1 44.8 %/G2 13.6 %; *p* = 0.031).

There were a few products with an indication of ideal pH for children's skin or acidic pH on the packaging of both groups (G1 31 %/G2 13.6 %; *p* = 0.192).

We also evaluated the possible allergens present in the composition of the moisturizers and observed that the most prevalent were fragrance/perfume and olfactory notes, as well as propylene glycol, pentylene glycol, and betaine. When comparing the groups, we were able to observe that the presence of these allergens was more prevalent in group 1 (G1 79.3 %/G2 45.5 %; *p* = 0.018), presenting statistical significance in this analysis. When we analyzed these components individually, the analyses with statistical significance were in relation to the presence of fragrance or perfume, which was present in 22 moisturizers in group 1 (75.9 %) and in 2 moisturizers in group 2 (9.1 %), and in relation to the presence of pentylene glycol, which was present in 6 moisturizers in group 2 (27.3 %) and none in group 1. The olfactory notes were present in 4 moisturizers in group 1 (13.8 %) and none in group 2. Propylene glycol was present in 3 moisturizers in group 1 (10.3 %) and 2 in group 2 (9.1 %). And betaine was present in 2 moisturizers in group 2 (9.1 %) and none in group 1. These last analyses did not show statistical significance between the groups.

## Discussion

The current trend of dermocosmetics that respect the pH of the skin surface and have a similar pH in their composition, being considered predominantly acidic, is extremely important, since they do not interfere as intensely with the skin microflora and have less harmful potential. Thus, in our critical evaluation study of several children's moisturizers, we were able to confirm that all the moisturizers tested have a pH lower than 7.0 and most of them are present in the pH range between 5.0 and 5.9.

When comparing moisturizers in two distinct groups - in terms of their nature, focused on serving the general pediatric public or with a therapeutic focus, we were able to observe that the members of the first group had an average pH of 5.81 ± 0.35, while the members of the second group had an average pH of 5.42 ± 0.28, with this difference being considered statistically significant (*p* ≤ 0.001). This predominance of a slightly more acidic pH in the second group may be relevant when we observe that it is closer to the pH of the skin's surface, being more compatible with maintaining its microbiome.

When analyzing the vehicle used in moisturizers, we were able to observe the preference of most products for the lotion vehicle in both groups, probably due to better spreadability of the product, providing greater user adherence in daily use, since many patients prefer a more fluid sensory experience in the application.^17^^,^^28^

Cost-benefit is essential when analyzing so many options for children's moisturizers since many are for daily use and need to be reapplied frequently.^17^^,^^28^ Group 1 stood out with a more affordable cost for the user, when compared to group 2, with statistical significance between them.

Regarding the analysis of the packaging of children's moisturizers, we were able to assess that in both groups the majority already present data such as the absence of dyes and potential endocrine disruptors in their components, such as parabens, phthalates and phenols, which is extremely important when evaluating dermocosmetics intended for children's use.

Although most moisturizers contain information on the packaging that they are “hypoallergenic”, we can observe a high frequency of allergens, such as perfume, in their composition, which demonstrates a lack of regulation of these products regarding the components described on the packaging. The predominance of fragrance/perfume among the components of the moisturizers in group 1 showed statistical significance in our analyses and the importance of this data is reflected in the fact that allergies to fragrances are the most common cause of cosmetic allergic contact dermatitis^28^ and, in fact, when analyzing the standard children's contact test battery, three of the twenty items tested refer to fragrance/perfume.^20^

In the children's moisturizers in the group focused to the general public, we observed a predominance of information on the packaging such as “natural ingredients”, “vegan ingredients” and “animal cruelty-free”. It is worth noting here that natural ingredients have become increasingly popular in recent years due to the growing awareness of environmental protection and animal welfare. In addition, natural oils and butters are often also more affordable.^29^ However, not all these ingredients are effective in maintaining skin integrity and can often cause further irritation. In fact, patients should be informed that products labeled as “natural” or “organic” may still contain plant extracts that can cause irritation or allergenicity and that these products may not necessarily be safer for the skin.^28^^,^^29^

The accepted definition of a vegan product is that it does not contain any animal products or by-products. Ingredients commonly excluded from vegan product formulations include lanolin, honey, beeswax, collagen, albumin, carmine, cholesterol, and gelatin.^29^

When we get to the subject of “cruelty-free” cosmetics, we can define them as those that do not participate in animal testing. This definition can be open to interpretation. For example, a final product may not be tested on an animal, but the ingredients contained in the product must have been tested on animals at the time of registration. In other words, the legislation requires testing only for new ingredients, and it is not necessary to carry out testing on formulations that include ingredients that have already been tested and formulated below the limiting concentrations. The Leaping Bunny Program and PETA (People for the Ethical Treatment of Animals) are widely recognized entities for certifying that several companies are free from animal testing at all stages of product development.^29^

In our analyses, we also found that few of the selected moisturizers had information on the labels related to the product's pH, only 11 of the 52 moisturizers evaluated (21 %), which may make it difficult for consumers to observe the relevance of dermocosmetics that respect the pH of the skin's surface for daily consumption, so it would be important for this data to be available on the product packaging.

It would also be relevant for the regulatory agencies that regulate the release of products for children's use to establish criteria for disclosing information contained on the packaging that has potential for marketing appeal, such as, for example, description of the presence of natural ingredients, since this is not necessarily related to the product's lower allergenicity. Although children's moisturizers have been exempt from registration with ANVISA since 2018, this does not reduce the technical requirements that must be met or the manufacturers' responsibility for these products.^30^

When evaluating the best cost-benefit of the moisturizers in this sample, we considered the items that we believe to be essential – such as pH range < 6.0, absence of potential allergenic components in the formulation and lowest cost per 100 mL of product. We observed that the moisturizers “Loção Hidratante Pampers Babytopia” belonging to G1 and “Hidratante Nutritivo Derma Protect Johnson’s Baby” belonging to G2 stand out, including the latter with an ideal pH range (<5), as shown in Table 3.

**Table 3:** Assessment of the best cost-benefit of the moisturizers present in this sample, considering the items that we believe to be essential – such as pH range < 6.0, absence of potential allergenic components in the formulation and organized in order from lowest to highest cost per 100 mL of product.

Best value for money assessment of sample moisturizers	pH	Cost per 100mL	Presence of possible allergens
Loção Hidratante Pampers Babytopia	5.62	29,95	No
Hidratante Nutritivo Derma Protect Johnson’s Baby	4.9	34,99	No
Loção Hidratante Corporal Isdin Para Pele Atópica Nutratopic Pro-AMP	5.75	40,99	No
Hidratante Calmante Para Pele Muito Sensível Mustela	5.62	44,99	No
Bruma Corporal Hidratante Hydraporin AI	5.58	50,52	No
Hidratante Corporal La Roche Posay Lipikar Baume Light AP+*M*	5.60	57,49	No
Creme Hidratante Mustela Bio	5.10	59,99	No
Loção Hidratante Infantil Hidra Kids D.A. Pele Sensível	5.4	63,65	No
Loção Hidratante Hydraporin AI	5.42	70,82	No
Loção Hidratante Umiditá Infantil Pele Sensível	5.56	71,65	No
Loção Hidratante CeraVe	5.45	79,98	No
Creme Hidratante CeraVe	5.56	79,98	No
NutriolMed Hidratante Intensivo Anticoceira Darrow	5.70	79,99	No
Hidratante Stelatopia + Relipidante Mustela	5.6	81,66	No
Creme Hidratante Reparador Pampers	5.75	156,33	No
Creme Reparador Hidratante Cicababy Boti Baby Boticário	5.67	216,33	No

Limitations: The selection of moisturizers did not cover all products on the market. Most of the moisturizers analyzed are produced and sold in Brazil. Although some of them represent internationally renowned brands, we cannot state that their chemical composition and, consequently, their pH are the same worldwide. We can also say that cosmetic products change their formulations quickly over time and this study analyzed moisturizers purchased within a predetermined period of time.

Despite this limitation, product brands were chosen that are also available in other countries, in easily accessible and popular establishments, and, furthermore, because we acquired the largest possible number of brands, we believe that our results can be extrapolated to other cities in Brazil and other regions of the world and should be corroborated by studies in these locations.

This study demonstrates that all moisturizers evaluated respected the slightly acidic pH suggested for maintaining the physiology of the skin surface, however, the group of moisturizers with a therapeutic focus on atopic children had an even lower pH and lower allergenic potential in their composition compared to the group of moisturizers focused on care of children with normal skin, despite the higher cost.

## Funding sources

This research did not receive any specific grant from funding agencies in the public, commercial, or not-for-profit sectors.

## Conflicts of interest

The authors declare no conflicts of interest.
